# Oncogenic KRAS sensitizes premalignant, but not malignant cells, to Noxa-dependent apoptosis through the activation of the MEK/ERK pathway

**DOI:** 10.18632/oncotarget.3552

**Published:** 2015-03-12

**Authors:** Annalisa Conti, Maria Teresa Majorini, Richard Elliott, Alan Ashworth, Christopher J. Lord, Carlotta Cancelliere, Alberto Bardelli, Pierfausto Seneci, Henning Walczak, Domenico Delia, Daniele Lecis

**Affiliations:** ^1^ Department of Experimental Oncology and Molecular Medicine, Fondazione IRCCS Istituto Nazionale dei Tumori, Milan, Italy; ^2^ The Breakthrough Breast Cancer Research Centre and CRUK Gene Function Laboratory, The Institute of Cancer Research, London, UK; ^3^ Department of Oncology, University of Torino, Candiolo, Torino, Italy; ^4^ Candiolo Cancer Institute - FPO, IRCCS, Candiolo, Torino, Italy; ^5^ FIRC Institute of Molecular Oncology (IFOM), Milano, Italy; ^6^ Università Degli Studi di Milano, Dipartimento di Chimica, Milan, Italy; ^7^ Centre for Cell Death, Cancer, and Inflammation, University College London, London, UK; ^8^ Current Address: UCSF Helen Diller Family Comprehensive Cancer Centre, San Francisco, California, USA

**Keywords:** KRAS, Smac mimetics, colorectal cancer, camptothecin

## Abstract

KRAS is mutated in about 20-25% of all human cancers and especially in pancreatic, lung and colorectal tumors. Oncogenic KRAS stimulates several pro-survival pathways, but it also triggers the trans-activation of pro-apoptotic genes. In our work, we show that G13D mutations of KRAS activate the MAPK pathway, and ERK2, but not ERK1, up-regulates Noxa basal levels. Accordingly, premalignant epithelial cells are sensitized to various cytotoxic compounds in a Noxa-dependent manner. In contrast to these findings, colorectal cancer cell sensitivity to treatment is independent of KRAS status and Noxa levels are not up-regulated in the presence of mutated KRAS despite the fact that ERK2 still promotes Noxa expression. We therefore speculated that other survival pathways are counteracting the pro-apoptotic effect of mutated KRAS and found that the inhibition of AKT restores sensitivity to treatment, especially in presence of oncogenic KRAS. In conclusion, our work suggests that the pharmacological inhibition of the pathways triggered by mutated KRAS could also switch off its oncogene-activated pro-apoptotic stimulation. On the contrary, the combination of chemotherapy to inhibitors of specific pro-survival pathways, such as the one controlled by AKT, could enhance treatment efficacy by exploiting the pro-death stimulation derived by oncogene activation.

## INTRODUCTION

KRAS is a 21 KDa protein involved in cell signal transduction belonging to the RAS subfamily, which comprises several other small GTPases endowed with GTP-hydrolyzing activity. In unstimulated conditions, GTPases are bound to GDP and display low activity, unable to trigger the down-stream signaling processes. RAS proteins require GTP to be activated and undergo rapid cycles of activation and inactivation crucial for physiological signaling [1]. Because these cascades stimulate cell growth and division, aberrant RAS signaling can also lead to cancer. The 3 human RAS genes (HRAS, KRAS, and NRAS) are among the most prevalent drivers of human cancer, with KRAS being mutated in 20-25% of all human tumors and up to 90% in certain cancer types, e.g. pancreatic cancer [2]. In these settings, KRAS activates several down-stream effectors leading to the stimulation of the RAF/mitogen-activated protein kinase kinase/extracellular signal-regulated kinase (RAF/MEK/ERK) and phosphatidylinositol-3-kinase (PI3K) pathways.

Colorectal cancer (CRC), one of the most widespread cancer types, displays in 40% of cases KRAS activating mutations, primarily involving codon 12 or 13. Several drug combinations are currently used for CRC treatment, including oxaliplatin, 5-FU and the camptothecin (CPT) analogue irinotecan [3]. Moreover, the epidermal growth factor receptor (EGFR)-blocking antibodies cetuximab and panitumumab are approved for treatment of metastatic CRC in combination with chemotherapy and as maintenance therapy in chemorefractory tumors. Receptor tyrosine kinases such as EGFR, through the activation of the downstream GTPases, regulate MAPK and PI3K pathways. Importantly, mutations or amplification of KRAS is often associated to unresponsiveness and acquired resistance to cetuximab [4].

Even though oncogenic KRAS is often associated with poorer prognosis, its mutations have also been considered for targeted therapy taking advantage of combinations that produce a synthetic lethal effect [5, 6]. In fact, the presence of constitutively active KRAS sensitizes cancer cells to MEK and BCL-XL [7] or RAF [8] inhibition, TRAIL [9], 5-FU and oxaliplatin [10]. Nonetheless, KRAS activation is usually associated with reduced proneness to apoptosis and increased resistance to chemotherapy owing to the activation of pro-survival pathways [11-13] and resulting in the up-regulation of anti-apoptotic factors such as the members of the inhibitor of apoptosis proteins (IAP) family [14, 15].

IAPs are characterized by the presence of a conserved baculoviral IAP repeat (BIR) domain [16] important for protein-protein interactions. Despite the 8 members of the IAP family had initially been considered essentially apoptosis negative regulators, only X-linked IAP (XIAP) is known to physically interact with caspases and prevent their activity [17]. Later studies have shown that IAPs regulate cell life aspects other than apoptosis. Cellular IAP1 (cIAP1) and cIAP2, for example, modulate the signaling of pro-survival pathways, such as the ones regulated by NF-kB transcription factors and MAPKs [16]. Interestingly, IAPs are often deregulated in cancer cells and associated to unfavorable prognosis [18]. An opportunity to target IAPs, and especially cIAP1, cIAP2 and XIAP, both for therapeutic purposes and as tools in pre-clinic research is represented by second mitochondria-derived activator of caspases (SMAC) mimetic (SM) small compounds [19]. SMs were designed to mimic the activity of SMAC [20], a natural antagonist of XIAP, which, by interacting with its BIR domains, displaces caspases and promotes their activity with consequent apoptosis induction. SMs also target cIAP1 and cIAP2, causing their degradation [21, 22], modulating several pathways and overcoming cancer cell resistance to therapy [23] and especially to tumor necrosis factor-related apoptosis inducing ligand (TRAIL) [20, 24].

Here we report that SM83, a SM recently described by us [25, 26], greatly enhances the cytotoxic activity of the topoisomerase I inhibitor CPT in premalignant models in which KRAS G13D is endogenously or ectopically expressed in human epithelial cells. The increased sensitivity of oncogenic KRAS-expressing cells stems at least in part from the basal up-regulation of the pro-apoptotic protein Noxa, which is stimulated in an ERK2-dependent manner. In clear contrast to the premalignant models, a panel of CRC lines with knock-in (KI) and knock-out (KO) mutations of KRAS G13D showed that the sensitivity to treatment is independent of KRAS status. Accordingly, Noxa levels are unaffected by oncogenic KRAS expression and other pathways, such as the ones controlled by PI3K/AKT, protect cancer cells from the potentially pro-apoptotic stimulus of mutated KRAS.

## RESULTS

### The combination of SMs and CPT selectively kills premalignant epithelial cells bearing oncogenic *KRAS*

**Figure 1:**
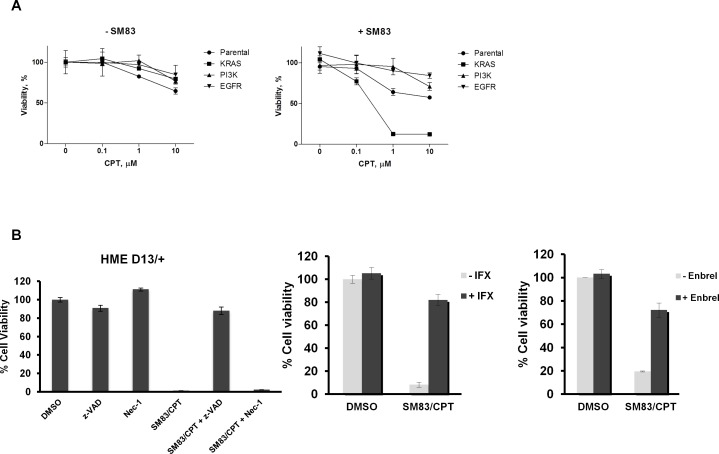

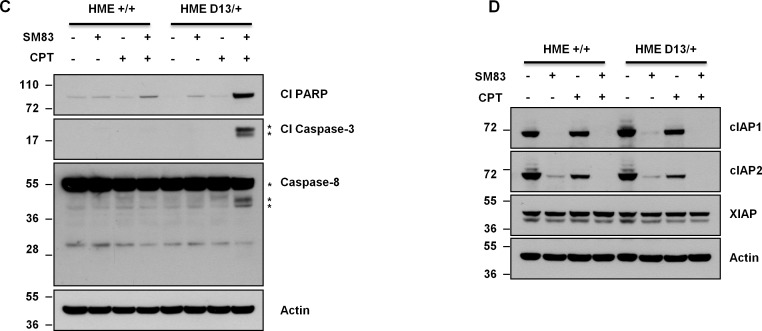
Oncogenic *KRAS* increases sensitivity of HME cells to DNA-damaging agents and TRAIL (A) The parental human epithelial (HME) cell line and the isogenic cell lines with knock-in mutations in KRAS (G13D), PI3K (H1047R) and EGFR (delE746A750) were treated with varying doses of CPT alone (left panel) or in combination with 100 nM SM83 (right panel). Viabilities are shown after 24 h of treatment. (B) HME D13/+ cells were pre-incubated with DMSO, 50 μM z-VAD, 20 μM Nec-1 (left panel), 10 μg/ml Infliximab (IFX, middle panel) and 10 μg/ml Enbrel (right panel) for 1 h and subsequently treated with 100 nM SM83 and 1 μM CPT. Cell viability was determined after 24 h. (C, D) HME +/+ and HME D13/+ cells were mock treated and treated with 100 nM SM83, 1 μM CPT and with their combination for 6 h. Cells were lysed and subjected to western blot to detect the apoptosis markers cleaved PARP, caspase-3 and caspase-8 (C) and the SM targets cIAP1, cIAP2 and XIAP (D). Actin is the loading control, asterisks show the cleaved forms p17/p19 of caspase-3 and the pro-caspase p55/p57 forms of caspase-8, together with its cleaved forms p41/p43. One representative of two independent experiments is shown.

As SMs are rarely effective in monotherapy, but sensitize cancer cells to other compounds, we searched for drugs whose cytotoxicity can be efficiently enhanced by SM83 using a high-throughput cell based screening approach. HeLa cells were exposed *in vitro* to SM83 and izTRAIL in addition to a combined library of about 3000 FDA-approved small molecule inhibitors and cell viability assessed (see Materials and Methods). Of the 3000 small molecule inhibitors assessed, we found that the topoisomerase I inhibitor camptothecin (CPT) most profoundly enhanced the cytotoxic effect of SM83 (Table 1). In addition to the enhancing effect of CPT, we also found that different formulations of CPT such as 10-hydroxycamptothecin also enhanced the effects of SM83, further confirming that CPT can be effectively combined with SMs and TRAIL. We then asked whether this combination is more cytotoxic in a specific genetic background and treated a panel of premalignant and cancer cell lines with izTRAIL, SM83 and CPT alone or in combination (data not shown). Viability tests showed that the immortalized human epithelial (HME) cell line bearing a KI G13D mutation in the KRAS gene (D13/+) is far more sensitive to SM83 plus CPT treatment compared to the parental HME or to HME carrying mutations activating PI3K and EGFR (Figure 1A). Moreover, HME D13/+ cells were more sensitive to izTRAIL alone or in combination with SM83 (Figure S1 upper panels), to the topoisomerase II inhibitor etoposide (ETO) and to neocarzinostatin (NCS), a DNA double strand break inducer (Figure S1 lower panel), suggesting a general enhanced sensitivity to cell death more than a specific mechanism favoring CPT-mediated death. Pre-treatment with pan-caspase inhibitor z-VAD strongly supports the idea that SM83/CPT treatment kills HME D13/+ cells through an apoptotic mechanism (Figure 1B left panel). In fact, the blocking of caspases resulted in almost complete protection from the treatment, while necroptosis inhibitor Necrostatin-1 (Nec-1) showed only a negligible effect. Importantly, as TNF is known to be a pivotal player in SM-mediated cell death, HME D13/+ were also pre-treated with the TNF-specific blockers Infliximab (Figure 1B middle panel) and Enbrel (Figure 1B right panel) which both remarkably rescued cells from the treatment, confirming the involvement of TNF in the SM83/CPT cell killing. Finally, by biochemical analysis we further confirmed that SM83 strongly increases the pro-apoptotic effect of CPT, as is evident from the substantial accumulation of cleaved PARP, caspase-8 and -3 (Figure 1C). Importantly, the altered sensitivity to treatment in cells with wild type or mutated *KRAS* did not stem from a diverse expression of the SM known targets cIAP1, cIAP2 and XIAP (Figure 1D), which are also depleted at the same level by SM83.

**Table 1:** Best hits from the high-throughput screening. HeLa cells were treated with FDA-approved drugs in combination with SM83 and izTRAIL. The most effective 10 compounds enhancers of the cytotoxic effect are listed

Compound	P-value
10-hydroxycamptothecin	0,000103319
Camptothecin	0,000040974
Camptothecine (S,+)	0,001697753
AMSACRINE	0,000274229
FLUOROURACIL	0,000537959
Aminacrine hydrochloride	0,015471379
Decitabine	0,000022640
MEFLOQUINE	0,000168791
Sutent	0,000444038
NETILMICIN SULFATE	0,005887782

### Endogenous and ectopic oncogenic *KRAS* sensitizes human epithelial cells to SM83 and CPT treatment

**Figure 2:**
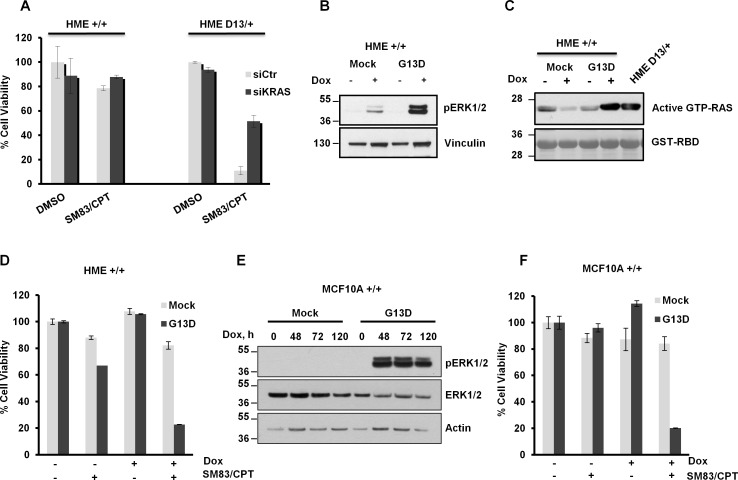
Endogenous and ectopic mutated *KRAS* confers sensitivity to SM83 and CPT co-treatment (A) HME +/+ and HME D13/+ were transfected with siRNA targeting KRAS for 48 h and subsequently treated with 100 nM SM83 and 1 μM CPT. Cell viability was determined after 24 h of treatment. (B) HME pINDUCER20-Mock (Mock) and HME pINDUCER20-KRAS G13D (G13D) were incubated with doxycycline (Dox, 250 ng/ml) for 48 h, lysed and a western blot was performed. The presence of activated KRAS was determined by detection of phosphorylated ERK1/2. (C) Active GTP-RAS was purified in cells stimulated as in (B) by pull-down assay using the recombinant RBD domain of RAF1; HME D13/+ are shown as positive control for activated KRAS. (D) HME Mock and KRAS G13D were incubated with Dox (250 ng/ml) for 48 h and treated with 100 nM SM83 and 1 μM CPT. Cell viability was determined after 24 h. (E) MCF10A Mock and KRAS G13D were incubated with Dox (250 ng/ml) for the indicated time, lysed and analyzed by western blot for the detection of ERK1/2 and phosphorylated ERK1/2. Actin is shown as a loading control. (F) MCF10A Mock and KRAS G13D were incubated with Dox (250 ng/ml) for 48 h and treated with 100 nM SM83 and 0.1 μM CPT. Cell viability was determined after 24 h. One representative of two independent experiments is shown.

To further investigate the role of mutated KRAS in the increased sensitivity of HME, the cytotoxic response to CPT and SM83 was assessed following total KRAS knockdown. The results showed that reduced KRAS decreased the toxicity by about 50% (Figure 2A), thus confirming the involvement of KRAS in the enhanced sensitivity. Unfortunately, the lack of an antibody specific for mutant KRAS did not allow us to determine the efficiency of G13D down-regulation (Figure S2). Furthermore, the silencing also affected wild type KRAS, which might also have a protective role to the treatment. To overcome this limit, KRAS G13D was inducibly expressed in HME cells using doxycycline. Augmented levels of phosphorylated ERK1/2 (Figure 2B), a down-stream effector of KRAS, and GST-RBD pull-down experiments confirmed the increased expression of activated KRAS (Figure 2C) paralleled by an hypersensitivity to SM83/CPT co-treatment (Figure 2D). We then repeated the experiments with another human epithelial cell line to exclude a possible cell line-specificity of our observation. MCF10A transduced with the KRAS G13D inducible vector confirmed that expression of mutant KRAS causes the phosphorylation of ERK1/2 (Figure 2E) and hypersensitivity to cell death (Figure 2F).

### Oncogenic KRAS-mediated up-regulation of Noxa sensitizes cells to SM83/CPT co-treatment

**Figure 3:**
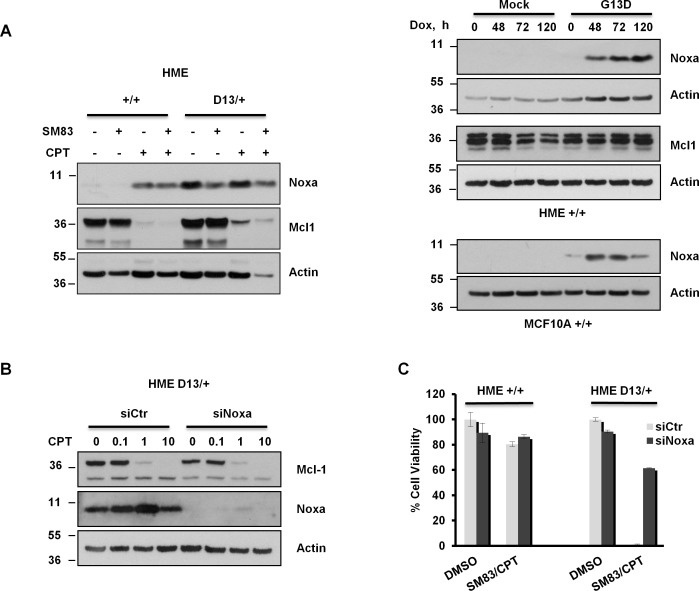
Increased Noxa expression in *KRAS*-mutated HME favours SM83/CPT-induced cell death (A, left panel) HME +/+ and HME D13/+ cells were treated with 100 nM SM83 and 1 μM CPT for 6 h, lysed and subjected to western blot to detect Noxa and Mcl1 levels. (A, right panel) HME/MCF10A Mock and KRAS G13D were incubated with Dox (250 ng/ml) for the indicated time and subjected to western blot to detect Noxa and Mcl1 levels. Actin is shown as the loading control. (B) HME D13/+ cells were trasfected with control and Noxa-targeting siRNAs and, after 48 h, were treated with the indicated concentration of CPT (μM) for 6 h. Cells were then lysed and analyzed by western blot to evaluate Mcl1 levels. Noxa is shown to check the silencing efficiency and actin as the loading control. (C) HME +/+ and HME D13/+ were transiently transfected with siRNA targeting Noxa for 48 h and subsequently treated with 100 nM SM83 and 1 μM CPT. Cell viability was determined after 24 h. One representative of two independent experiments is shown.

To determine the mechanisms by which oncogenic KRAS sensitizes non-tumoral cells to treatment, several cell lines expressing endogenous and ectopic KRAS G13D were analyzed by western blot for the levels of pro- and anti-apoptotic proteins of the Bcl-2 family (data not shown). In accordance to other works, we found that the presence of oncogenic KRAS considerably increases the basal levels of Noxa in untreated cells. Accordingly, HME bearing the KI G13D mutation displayed higher levels of Noxa compared to the parental cell line (Figure 3A left panel), while the basal levels of the Noxa natural antagonist Mcl-1 were not affected by oncogenic KRAS expression, but markedly dropped after CPT treatment in a SM83-independent manner. Moreover, transient induction of ectopic KRAS G13D in HME and MCF10A parental cell lines concurred to a marked increase of Noxa levels in both cell lines (Figure 3A right panels, upper and lower panel respectively). In line with these data, KRAS silencing reduced the levels of Noxa in HME KRAS G13D cells (Figure S2). Furthermore, since Mcl1 levels were reduced concurrently to Noxa up-regulation in HME cells (Figure 3A left panel), we checked whether Noxa increase was responsible for Mcl1 down-regulation. Mcl1 levels were therefore detected in KRAS G13D-induced HME cells which showed that the sole Noxa up-regulation in not sufficient to affect Mcl1 levels in untreated cells. We then analyzed by western blot HME KI D13/+ cells silenced with control or Noxa-specific siRNAs and treated with increasing concentrations of CPT (Figure 3B). Also in this case Mcl-1 stability was independent of Noxa presence, suggesting that Mcl-1 down-regulation stems from the treatment and not from Noxa up-regulation. To determine whether the oncogenic KRAS-dependent accumulation of Noxa is responsible for the hypersensitivity to SM83/CPT, viability tests were performed after Noxa depletion (Figure S2). The results showed that Noxa silencing confers resistance to treatment in HME D13/+ cells (Figure 3C).

### KRAS-induced up-regulation of Noxa is mediated by ERK2

**Figure 4:**
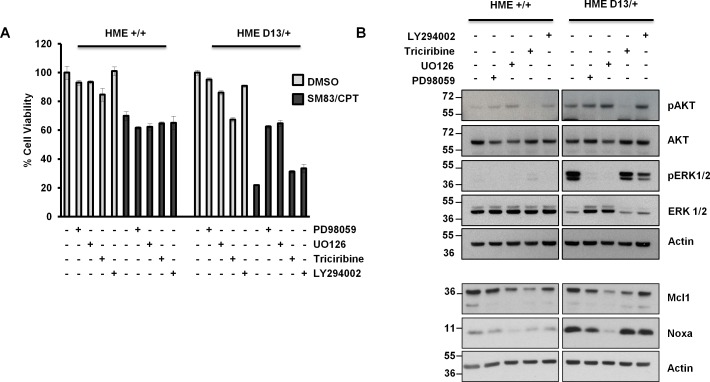

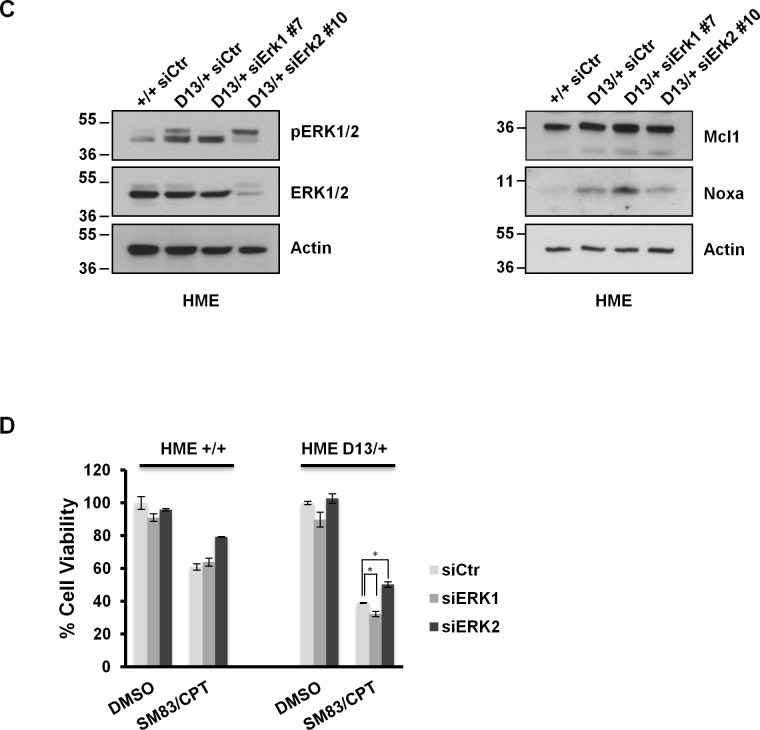
ERK2, but not ERK1 is responsible for KRAS-dependent Noxa-induction (A) HME +/+ and HME D13/+ cell lines were pre-incubated with 50 μM PD98059, 25 μM UO126, 20 μM Triciribine and 20 μM LY294002 for 2 h, and then treated with 100 nM SM83 and 1 μM CPT. Cell viability was quantified after 24 h. One representative of three independent experiments is shown. (B) HME +/+ (left panel) and HME D13/+ (right panel) cell lines were treated with 50 μM PD98059, 25 nM UO126, 20 μM Triciribine and 20 μM LY294002 for 2 h, and subsequently analyzed by western blot to detect the phosphorylated forms of AKT, ERK1 and ERK2, their total levels (upper panels) or Noxa and Mcl1 (lower panels). Actin is shown as loading control. (C) HME +/+ and HME D13/+ were transiently transfected with the indicated siRNAs for 72 hours and subsequently analyzed by western blot to detect total and phosphorylated ERK1 and ERK2, and their total levels (left panel), Noxa and Mcl1 (right panel). Actin is shown as loading control. (D) Parental HME +/+ and HME D13/+ cells were silenced for 48 h and then treated with DMSO and 100 nM SM83 plus 1 μM CPT for further 24 h. One representative of three independent experiments is shown. * *P* < 0.05 vs siCtr.

We next investigated the mechanisms responsible for the acquired sensitivity of KRAS-mutated HME to treatment. Both parental and D13/+ HME cell lines were treated with CPT and SM83 in the presence of various inhibitors of the MAPK, AKT and PI3K pathways which can be stimulated by activated RAS. In parental cells, the administration of MEK1/2 inhibitors PD98059 and UO126, AKT inhibitor Triciribine or PI3K inhibitor LY294002 did not affect significantly the toxicity of SM83/CPT treatment (Figure 4A). In contrast, both MEK1/2 inhibitors partially protected D13/+ HME cells from SM83/CPT treatment and conferred resistance at the same degree as parental cells (Figure 4A). Having found that Noxa is a pivotal mediator of KRAS-dependent increased sensitivity to the combination (Figure 3C), we evaluated whether the MAPK pathway was responsible for the increased levels of Noxa. We found that both MEK inhibitors reduced, as expected, the levels of phosphorylated ERK1 and ERK2, and concurrently reduced the levels of Noxa (Figure 4B). Interestingly, also Mcl1 levels were slightly reduced by the MEK inhibitors, suggesting that both Noxa and Mcl1 expression is regulated by the MEK/ERK pathway. Importantly, MEK inhibition slightly reduced Noxa basal levels also in parental HME (left panel) suggesting that the MAPK pathway stimulates Noxa also in physiological conditions. To understand whether MEK targets ERK1 and ERK2 both contribute to Noxa regulation, we silenced each of them in D13/+ HME cells and found that only ERK2 down-regulation reduced Noxa levels, while ERK1 silencing marginally increased accumulation of Noxa (Figure 4C right panel). Again, Mcl1 was not down-regulated by Noxa accumulation, further confirming that the treatment with CPT, and not Noxa up-regulation, was responsible for Mcl1 reduction in HME cells (Figure 3A). In line with the regulation of Noxa observed in Figure 4C, ERK1 silencing slightly, but significantly, enhanced the sensitivity of D13/+ HME cells to SM83/CPT treatment, while ERK2 silencing resulted in the opposite effect (Figure 4D).

### Sensitivity to SM83/CPT is independent of KRAS status in a panel of colorectal cancer cell lines

**Figure 5:**
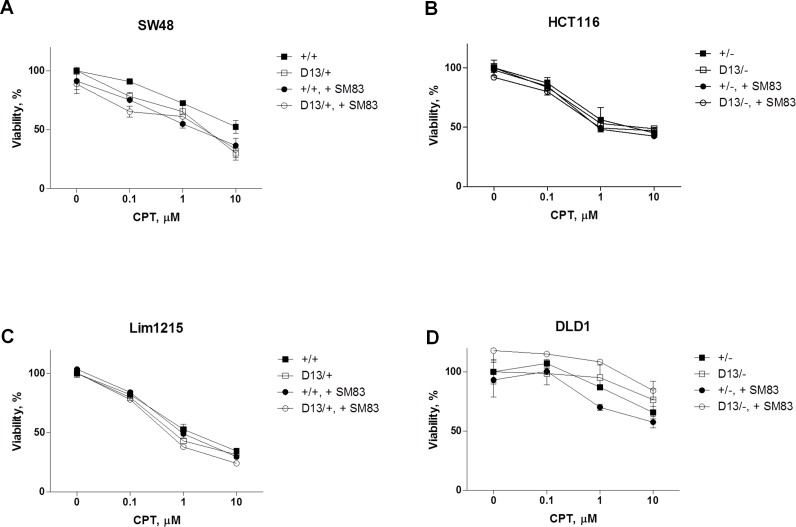

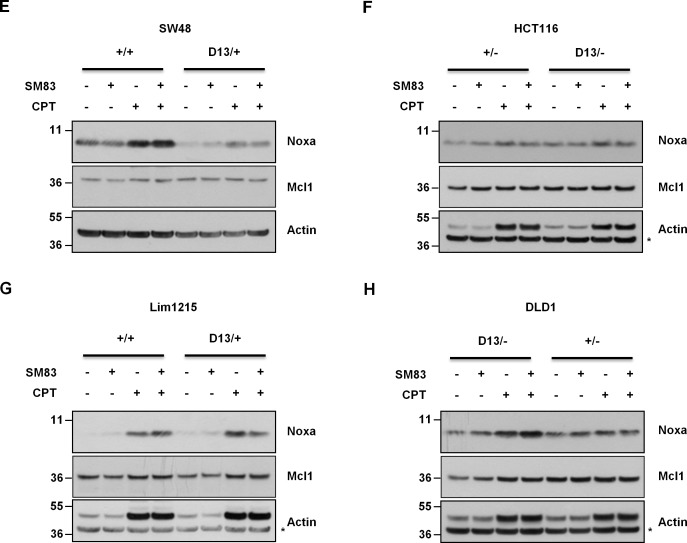
Oncogenic mutation of *KRAS* does not confer sensitivity to combined SM83/CPT nor stimulates Noxa levels SW48 +/+ and SW48 D13/+ (A), HCT116 +/− and HCT116 D13/− (B), Lim1215 +/+ and Lim1215 D13/+ (C), DLD1 D13/− and DLD1 +/− (D) cell lines were treated with DMSO and the combination of SM83 and CPT at varying concentrations. Cell viability was evaluated after 24 h. One representative of three independent experiments is shown. SW48 +/+ and SW48 D13/+ (E), HCT116 +/− and HCT116 D13/− (F), Lim1215 +/+ and Lim1215 D13/+ (G), DLD1 D13/− and DLD1 +/− (H) cell lines were treated with DMSO and 100 nM SM83, 0.1 μM CPT either alone or in combination for 6 h. Cells were lysed and analyzed by western blotting to determine Noxa and Mcl1 levels. One representative of two independent experiments is shown. Asterisk indicates the specific band of Actin shown as loading control.

Our findings support the notion that oncogenic KRAS can sensitize premalignant cells to SM83/CPT treatment. We then considered whether this also occurs in malignant cells, and for this reason we employed a panel of isogenic CRC cell lines where mutated KRAS is either KI (+/+ and D13/+, SW48 and Lim1215) or KO (D13/− and +/−, HCT-116 and DLD1). Surprisingly and in contrast to the premalignant settings, the sensitivity of the CRC cell lines to SM83/CPT treatment was independent of the KRAS status (Figure 5A-D). We then investigated the Noxa status, which was responsible for the increased sensitivity of normal epithelial cells bearing oncogenic KRAS, and found that its levels were unaffected by the presence of mutated KRAS (Figure 5E-H). Likewise, the levels of Noxa-antagonist Mcl1 were not repressed by the treatment with CPT and/or SM83, and further experiments confirmed an increased stability of Mcl1 in colorectal cancer compared to HME cells (Figure S3). Noxa basal levels were however higher in CRC than in HME cell lines (data not shown), suggesting that pro-apoptotic mediators can even be up-regulated in tumor cells, but there are likely other activated pathways that counterbalance the potential pro-apoptotic stimuli.

### Aberrant activation of AKT counterbalances KRAS-mediated pro-apoptotic scenario in colorectal cancer cells

**Figure 6:**
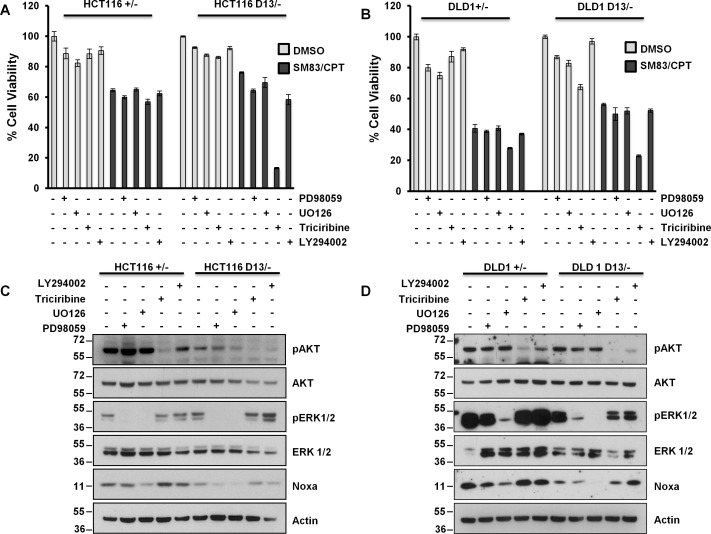

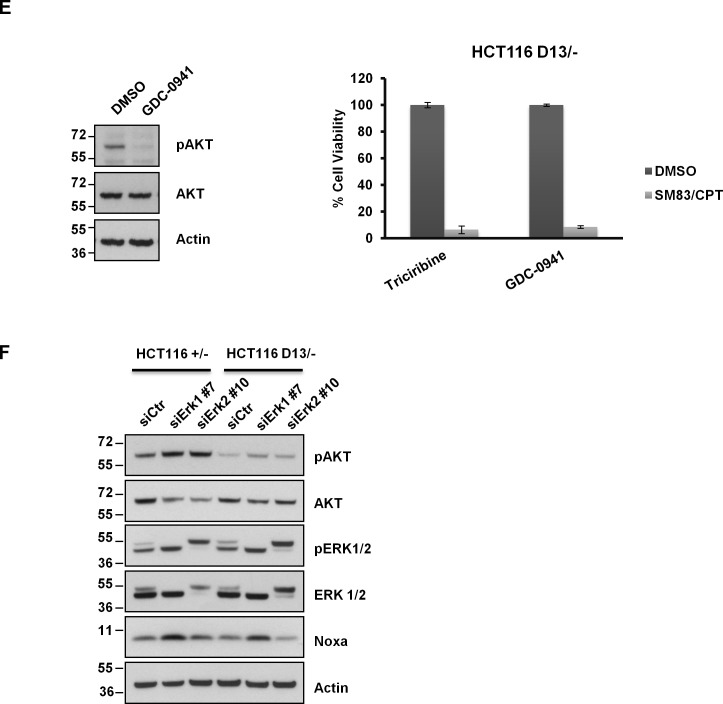
Aberrant AKT activation protects HCT116 and DLD1 cells from the pro-death effect of oncogenic *KRAS* (A) HCT116 and (B) DLD1 cells were pre-incubated with 50 μM PD98059, 25 μM UO126, 20 μM Triciribine and 20 μM LY294002 for 2 h, and then mock-treated or treated with 100 nM SM83 and 1 μM CPT. Cell viability was quantified after 24 h. (C) HCT116 and (D) DLD1 cells were treated for 2 h with the indicated inhibitors as in (A) and then analyzed by western blot to detect Noxa levels and total and phosphorylated AKT and ERK levels. Actin is shown as loading control. (E, left panel) HCT116 D13/− cells were treated with 1 μM GDC-0941 for 2 h and analyzed by western blot to detect total and phosphorylated levels of AKT. Actin is shown as the loading control. (Right panel) Viability of HCT116 D13/− pre-treated with 1 μM GDC-0941 for 2 h and then treated with 100 nM SM83 and 1 μM CPT. Cell viability was quantified after 24 h and expressed as viability percentage to inhibitor alone. (F) HCT116 cells were transfected with siRNAs targeting ERK1 and ERK2, cells were collected after 72 h and analyzed by western blot to detect Noxa and total and phosphorylated levels of AKT and ERK. Actin is shown as loading control.

Despite the presence of mutant KRAS, our findings suggest that cancer cells are not sensitized to SM83/CPT treatment. Therefore, we hypothesized that malignant progression might have caused the deregulation of other pathways that can counterbalance the potential apoptotic effect of oncogenic KRAS. Interestingly, HCT116 and DLD1 cells bear mutated PI3K, which results in hyper-activation of the PI3K/AKT pathway, a signaling cascade known to promote cell survival. For this reason, we treated HCT116 and DLD1 cells bearing mutated and wild type KRAS with SM83/CPT after pre-treatment with inhibitors of MEK1/2, AKT and PI3K. Interestingly, and concordant to our hypothesis, AKT inhibition restored sensitivity to the treatment only in the presence of mutant KRAS (Figure 6A and 6B). Noxa levels were lowered by MAPK blocking (Figure 6C and 6D) as already observed in HME cells (Figure 4B), but were not affected by AKT inhibition, suggesting that the AKT pathway blocks the pro-death effect triggered by oncogenic KRAS in an independent fashion. Importantly, AKT inhibition sensitized to SM83/CPT treatment also CRC cell lines bearing wild type PI3K (Figure S4), further supporting the idea that AKT counteracts the pro-death stimulus deriving from oncogenic KRAS. Surprisingly, LY294002 treatment, which reduced AKT activation, did not sensitize to SM83/CPT treatment (Figure 6C and 6D). We speculate that this stems from the fact that the LY294002 inhibitor did not completely abolish AKT phosphorylation and therefore we tested the GDC-0941 PI3K inhibitor. This compound reduced the AKT activation more efficiently (Figure 6E left panel) and sensitized the HCT116 D13/− in the same way as Triciribine (Figure 6E right panel). Finally, we investigated the mechanisms by which Noxa levels are controlled in HCT116 cells and demonstrated that the findings described for HME are true also in this cancer cell line. In fact, the targeting of ERK1 by silencing enhanced the levels of Noxa, while a specific siRNA targeting ERK2 slightly reduced its expression (Figure 6F).

## DISCUSSION

In our work, we searched for FDA-approved drugs that increase the cytotoxic activity of IAP-antagonizing compounds and death ligands. For this purpose, using a high-throughput approach, we combined SM83 [25] and izTRAIL [29] to a library of about 3000 compounds. CPT was identified several times among the best hits and validated, alone or in combination with SM83 and/or izTRAIL, in a panel of normal and cancer cell lines bearing KI and KO mutations in genes frequently mutated in cancer. The employment of isogenic cell lines with distinct point mutations is a powerful tool to comprehend the effect of oncogene activation [31] and addiction [32], and synthetic lethal interactions [27, 33] in cancer cells. With this approach, we found that the endogenous and ectopic expression of KRAS bearing the G13D mutation sensitizes normal, but not cancer cells, to CPT plus SM83 or TRAIL treatment, and to other DNA-damaging agents.

Since oncogenic KRAS stimulates the up-regulation of the pro-apoptotic protein Noxa [10, 34], we checked the occurrence of this event in premalignant cells and whether it was associated with the increased sensitivity to treatment. In both human epithelial cells HME and MCF10A, the expression of oncogenic KRAS was indeed responsible for the up-regulation of Noxa in a MEK/ERK-dependent manner and for the augmented death upon SM83/CPT treatment. Accordingly, chemical inhibition of the MEK1/2 kinases that results in prevention of ERK1/2 phosphorylation and silencing of ERK2, but not ERK1, down-regulated Noxa in HME D13/+ to levels comparable to parental HME (+/+) cells. Of note, ERK2 silencing slightly, but reproducibly, protected HME D13/+ cells, while ERK1 silencing even sensitized to SM83/CPT treatment and simultaneously increased Noxa basal levels especially in CRC cell lines. The observation that ERK1 and ERK2 display opposite effects in regulating Noxa levels and mediating chemotherapy responsiveness was also described in hepatocellular carcinoma cells [35]. In this case, ERK2 knockdown was responsible for increased Noxa levels after cisplatin treatment. Although we investigated Noxa basal levels in our experiments, these contrasting results strongly support that ERK1 and ERK2 mutually regulate each other [36] in a cell type-dependent manner. Surprisingly, MEK inhibitors strongly prevented treatment cytotoxicity, while siRNA targeting ERK1 and ERK2 only have a modest effect, despite siERK2 efficiently down-regulated Noxa levels. This suggests that other unknown regulatory mechanisms between the two ERK proteins eventually impact on the treatment outcome.

**Figure 7:**
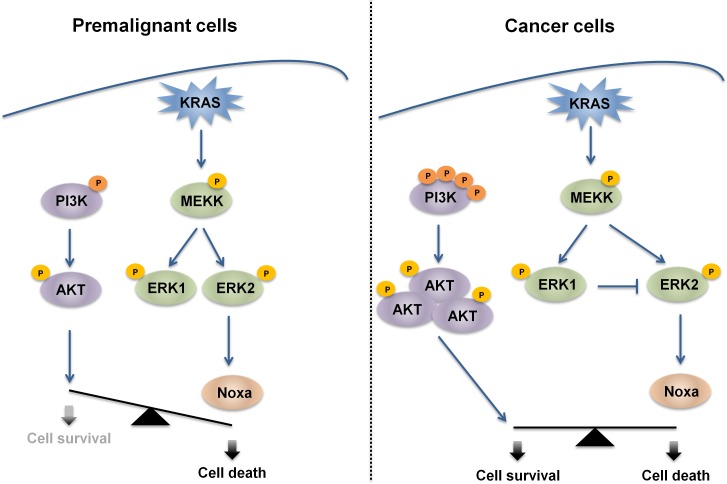
Proposed mechanism for oncogenic KRAS-mediated sensitization to cell death In premalignant models, normal epithelial cells expressing endogenous or ectopic mutated KRAS express high levels of Noxa due to the hyper-activation of MAPK kinases and in particular of ERK2. In these settings, the basal activation of the PI3K/AKT pathway is not sufficient to protect from this pro-death stimulus and treatment with several cytotoxic agents results in Noxa-dependent cell killing. In contrast, in CRC cells, Noxa levels are independent of KRAS status and oncogenic KRAS-bearing cells respond to treatment to the same extent as in the presence of wild-type KRAS. In fact, mutated PI3K and up-stream stimuli likely deriving from the tyrosine kinase receptors activate AKT, which counterbalances the potential pro-death stimulus deriving from oncogenic KRAS.

We then asked whether our findings are true not only in premalignant cells, but also in cancer cells. To this end, a panel of isogenic colon cancer cell lines with KI and KO mutations of KRAS was tested. In clear contrast to HME and MCF10A cells, Noxa levels did not depend on KRAS status in cancer cells and in line with this observation cell sensitivity was almost identical in each pair of isogenic cells. Basal levels of Noxa in cancer cells were higher than in epithelial cells (data not shown), suggesting that tumor cells constitutively express some pro-apoptotic proteins at high level, but could also activate parallel pro-survival pathways to counteract the pro-death signals supported by the MEK/ERK axis. Considerable evidence shows that mutations in the RAS/MEK/ERK cascade are associated to aberrant activation of PI3K/AKT signaling [37] and therefore both pathways should be targeted simultaneously for effective responsiveness to treatment [11, 38]. In accordance to this hypothesis, HCT116 cells and DLD1, which bear PI3K activating mutations, are sensitized to SM83/CPT treatment when pre-treated with AKT inhibitors only in the presence of oncogenic KRAS, supporting the notion that AKT is protecting from oncogenic KRAS-dependent cancer cell sensitization (Figure 7). It is important to note that this protective role was demonstrated also in cells bearing wild type PI3K (Figure S4), confirming the general pivotal role of AKT in counterbalancing the pro-death effect of oncogenic KRAS.

In conclusion, our work has two main implications. First, targeting down-stream effectors of oncogenes might result in an immediate and transient anti-proliferative effect often achieved by conventional therapies, but, more importantly, could also shut-down the pro-death signals derived from oncogene activation. Secondly, for a successful treatment, targeting of the EGFR/MAPK pathway alone is not sufficient [39], as it results in emerging protecting mutations [4, 40], feedbacks [41] and incomplete responses. It is therefore imperative to characterize and inhibit also the aberrantly activated survival pathways in a combination treatment, in order to overcome the anti-apoptotic effect of PI3K/AKT activation and simultaneously to exploit the pro-death signal stemming from oncogenic activation.

## METHODS

### Cell lines

The human isogenic hTERT-immortalized mammary epithelial cell lines HME +/+ and HME D13/+, and the human epithelial mammary MCF10A together with the isogenic pairs of colorectal cancer cell lines SW48 +/+ and SW48 D13/+, HCT116 +/− and HCT116 D13/−, Lim1215 +/+ and Lim1215 D13/+, DLD1 D13/− and DLD1 +/− have already been described [4, 27]. HME isogenic pairs and MCF10A cell lines were cultured in DMEM-F12 (Gibco), supplemented with 10% FBS (LONZA), 2 mM glutamine (LONZA), 20 ng/ml EGF (Immunological Science), 10 μg/ml insulin (Sigma), 500 μg/ml hydrocortisone (Sigma-Aldrich). SW48 and DLD1 isogenic pairs were cultured with DMEM (Gibco) supplemented with 10% FBS and 2 mM glutamine. Lim1215 isogenic pairs were cultured with RPMI (LONZA) supplemented with 10% FBS, 2 mM glutamine and 1 μg/ml insulin. HCT116 were cultured in RPMI, supplemented with 2 mM glutamine, sodium pyruvate (LONZA), and non-essential amino acids (LONZA). HeLa cells for the high-throughput screening and packaging HEK293FT (Life Technologies) for lentiviral production were cultured in DMEM with 10% FBS. All cell lines were mycoplasma-free as determined by Takara Mycoplasma Detection Kit (Clontech).

### Reagents

Antibodies targeting pan-RAS, Noxa (CalBiochem), Actin and ERK1/2 (Sigma), cleaved-PARP, cleaved caspase-3, phosphoERK1/2 (Thr202/Tyr204), pAKT and AKT (Cell Signaling), cIAP1 (R&D Systems), cIAP2 and XIAP (BD Biosciences), caspase-8 (Enzo Life Sciences) and Mcl-1 (Santa Cruz Biotechnology) were employed in western blot experiments. z-VAD(OMe)-FMK was purchased by BIOMOL, Necrostatin-1 from Enzo Life Sciences. PD98059 and UO126 were purchased from CalBiochem, LY294002 from Sigma, GDC-0941 and Triciribine from Selleckem. Infliximab (Schering-Plough) and Enbrel (Wyeth Pharmaceuticals) were used as TNF blockers. CPT and neocarzinostatin were purchased from Sigma-Aldrich, etoposide by Teva. SM83 synthesis has been described elsewhere [25, 28], while izTRAIL was purified as already shown [29]. Mutant KRAS (G13D) was cloned in the pINDUCER20 and lentiviral particles prepared modifying an already described protocol [30] and using Lipofectamine 2000 as transfection reagent. Expression of the transgene was induced by doxycycline (Sigma-Aldrich) at the indicated concentrations.

### High-throughput screening

On day 1, 350 HeLa cells/well were seeded in 384-well white plates in 20 μl medium. At day 2, media was changed with cells being exposed to 100 nM SM83 in addition to FDA-approved drug libraries (ENZO Life Sciences, MicroSource Discovery Systems Inc. and Prestwick Chemical, France) with the drug library compounds present at a final concentration of 1 μM. At day 3, cells exposed to SM83 were also exposed to 20 pg/ml izTRAIL. Cell viability was estimated on day 5 by CellTiter-Glo (Promega). Hits were selected due to their capability to enhance the cytotoxic activity of SM83/izTRAIL and then validated using the same HeLa cells employed in the screening. In validation experiments, SM83 and izTRAIL were administered alone and in combination, also changing the schedule and pre-treating cells with SM83 24 h before izTRAIL or administrating these compounds simultaneously. On the basis of these results, validated hits were tested in a panel of premalignant and cancer cell lines.

### Western blot

Cells were harvested by centrifugation at 4500 rpm for 5 min at 4°C. After washing once with PBS, lysates were prepared by resuspending cell pellets in 60-100 μl lysis buffer (125 mM Tris HCl pH 6.8, 5 % SDS) supplemented with 1x complete protease (Roche Diagnostics) and phosphatase inhibitors (Sigma-Aldrich). Lysates were boiled at 99°C, sonicated for 20 seconds at RT. Then lysates were centrifuged at 13000 rpm for 20 min at RT and cleared supernatants were transferred into a new tube and frozen at −20°C. Cell lysates were mixed with 4x reducing SDS-Sample buffer containing 10% β-mercaptoethanol (Sigma-Aldrich) and heated for 10 min at 99°C. Proteins (50 μg) were separated by SDS-PAGE using pre-cast 4-12% BisTris NuPAGE gels (Life Technologies), blotted to PVDF membranes (Millipore), which were washed with PBS-tween for 5 min, blocking buffer made of 4% non-fat milk in PBS-tween for 30 min and then incubated overnight with the indicated primary antibodies. Proteins were detected after hybridization with appropriate horseradish peroxidase (hrp)-conjugated secondary antibodies by adding a chemiluminescent substrate (EuroClone).

### Cell viability assays

96-well optical bottom, polymer base white plates (Thermo Scientific) were used for viability tests. At day 1, 10000 cells per well were seeded in 100 μl medium. At day 2, cells were treated adding the indicated drug(s) in 10 μl volume per well. At day 3, cell viability was determined using the CellTiter-Glo assay according to the manufacturer's instructions. Statistical analysis was performed with GraphPad Prism 5.02 using the two-tailed unpaired t-test.

### Transfection and lentiviral transduction

To achieve transient knock-down of target proteins in HME cells, a reverse transfection protocol employing siRNAs (Qiagen) and RNAiMAX (Life technologies) was used. Briefly, 3.25 μl RNAiMAX and 200 μl Optimem (GIBCO) were mixed and incubated for 5 min at RT. Subsequently, 3.25 μl siRNA of a 20 μM stock were added, mixed and incubated for further 30 min at RT. The transfection mix was placed in a 6-well plate and 0.15 × 10^6^ cells seeded in 800 μl on top.

Tumor cells were seeded the day before transfection and the same transfection mix as for reverse transfection was added on top of cells 24 h later. The cells were then incubated for 48 h before being drug-treated for further 24 h or cultured for 72 h before stopping the experiment. In each experiment, scramble siRNAs (siCtr) were used as a control.

Cells transduced with lentiviral particles were cultured in the presence of medium collected from HEK293FT packaging cells transfected with pINDUCER20-KRAS G13D (referred to as G13D) or empty vector (Mock). After 48 h, medium was replaced and fresh medium added in the presence of 500 μg/ml G418 (Life Technologies).

### Ras-GTP pull-down assay

2.5 × 10^6^ cells were seeded into 10 cm dishes. The next day, cells were incubated with and without 250 ng/ml of Dox. Cell lysis and RAS-GTP pulldown was performed. Cells were lysed in 500 μl of IP-lysis buffer supplemented with a cocktail of protease inhibitors. To fully detach lysed cells, they were scratched using a cell scraper and transferred into tubes for a 30-minute lysis at 4 °C on a rotator. Lysates were centrifuged at 13000 rpm for 30 min and cleared supernatants were transferred to a new tube. RAS-GTP was precipitated using beads coated with the RAF1-binding domain RBD recombinant protein. The following day, beads were washed 5 times with IP-lysis buffer and precipitated protein complexes were eluted from the beads via boiling in SDS-Sample buffer for 10 minutes at 80°C. Proteins were separated by SDS-PAGE and analyzed by western blot. As a loading control, proteins were stained by blue coomassie (Thermo Scientific Pierce).

## SUPPLEMENTARY MATERIALS AND FIGURES



## References

[1] Pylayeva-Gupta Y, Grabocka E, Bar-Sagi D (2011). RAS oncogenes: weaving a tumorigenic web. Nat Rev Cancer.

[2] Eser S, Schnieke A, Schneider G, Saur D (2014). Oncogenic KRAS signalling in pancreatic cancer. Br J Cancer.

[3] Meyerhardt JA, Mayer RJ (2005). Systemic therapy for colorectal cancer. N Engl J Med.

[4] Misale S, Yaeger R, Hobor S, Scala E, Janakiraman M, Liska D, Valtorta E, Schiavo R, Buscarino M, Siravegna G, Bencardino K, Cercek A, Chen CT (2012). Emergence of KRAS mutations and acquired resistance to anti-EGFR therapy in colorectal cancer. Nature.

[5] Weidle UH, Maisel D, Eick D (2011). Synthetic lethality-based targets for discovery of new cancer therapeutics. Cancer Genomics Proteomics.

[6] Steckel M, Molina-Arcas M, Weigelt B, Marani M, Warne PH, Kuznetsov H, Kelly G, Saunders B, Howell M, Downward J, Hancock DC (2012). Determination of synthetic lethal interactions in KRAS oncogene-dependent cancer cells reveals novel therapeutic targeting strategies. Cell Res.

[7] Corcoran RB, Cheng KA, Hata AN, Faber AC, Ebi H, Coffee EM, Greninger P, Brown RD, Godfrey JT, Cohoon TJ, Song Y, Lifshits E, Hung KE (2013). Synthetic lethal interaction of combined BCL-XL and MEK inhibition promotes tumor regressions in KRAS mutant cancer models. Cancer Cell.

[8] Lamba S, Russo M, Sun C, Lazzari L, Cancelliere C, Grernrum W, Lieftink C, Bernards R, Di Nicolantonio F, Bardelli A (2014). RAF suppression synergizes with MEK inhibition in KRAS mutant cancer cells. Cell Rep.

[9] Huang S, Ren X, Wang L, Zhang L, Wu X (2011). Lung-cancer chemoprevention by induction of synthetic lethality in mutant KRAS premalignant cells in vitro and in vivo. Cancer Prev Res (Phila).

[10] de Bruijn MT, Raats DA, Hoogwater FJ, van Houdt WJ, Cameron K, Medema JP, Borel Rinkes IH, Kranenburg O (2010). Oncogenic KRAS sensitises colorectal tumour cells to chemotherapy by p53-dependent induction of Noxa. Br J Cancer.

[11] Di Nicolantonio F, Arena S, Tabernero J, Grosso S, Molinari F, Macarulla T, Russo M, Cancelliere C, Zecchin D, Mazzucchelli L, Sasazuki T, Shirasawa S, Geuna M (2010). Deregulation of the PI3K and KRAS signaling pathways in human cancer cells determines their response to everolimus. J Clin Invest.

[12] Tao S, Wang S, Moghaddam SJ, Ooi A, Chapman E, Wong PK, Zhang DD (2014). Oncogenic KRAS confers chemoresistance by upregulating NRF2. Cancer Res.

[13] Hata AN, Yeo A, Faber AC, Lifshits E, Chen Z, Cheng KA, Walton Z, Sarosiek KA, Letai A, Heist RS, Mino-Kenudson M, Wong KK, Engelman JA (2014). Failure to induce apoptosis via BCL-2 family proteins underlies lack of efficacy of combined MEK and PI3K inhibitors for KRAS-mutant lung cancers. Cancer Res.

[14] Hadj-Slimane R, Pamonsinlapatham P, Herbeuval JP, Garbay C, Lepelletier Y, Raynaud F (2010). RasV12 induces Survivin/AuroraB pathway conferring tumor cell apoptosis resistance. Cell Signal.

[15] Moller Y, Siegemund M, Beyes S, Herr R, Lecis D, Delia D, Kontermann R, Brummer T, Pfizenmaier K, Olayioye MA (2014). EGFR-Targeted TRAIL and a Smac Mimetic Synergize to Overcome Apoptosis Resistance in KRAS Mutant Colorectal Cancer Cells. PLoS One.

[16] Gyrd-Hansen M, Meier P (2010). IAPs: from caspase inhibitors to modulators of NF-kappaB, inflammation and cancer. Nat Rev Cancer.

[17] Eckelman BP, Salvesen GS, Scott FL (2006). Human inhibitor of apoptosis proteins: why XIAP is the black sheep of the family. EMBO Rep.

[18] Fulda S (2014). Molecular pathways: targeting inhibitor of apoptosis proteins in cancer--from molecular mechanism to therapeutic application. Clin Cancer Res.

[19] Fulda S, Vucic D (2012). Targeting IAP proteins for therapeutic intervention in cancer. Nat Rev Drug Discov.

[20] Li L, Thomas RM, Suzuki H, De Brabander JK, Wang X, Harran PG (2004). A Small Molecule Smac Mimic Potentiates TRAIL- and TNF{alpha}-Mediated Cell Death. Science.

[21] Vince JE, Wong WW, Khan N, Feltham R, Chau D, Ahmed AU, Benetatos CA, Chunduru SK, Condon SM, McKinlay M, Brink R, Leverkus M, Tergaonkar V (2007). IAP Antagonists Target cIAP1 to Induce TNFα-Dependent Apoptosis. Cell.

[22] Varfolomeev E, Blankenship JW, Wayson SM, Fedorova AV, Kayagaki N, Garg P, Zobel K, Dynek JN, Elliott LO, Wallweber HJA, Flygare JA, Fairbrother WJ, Deshayes K (2007). IAP Antagonists Induce Autoubiquitination of c-IAPs, NF-κB Activation, and TNFα-Dependent Apoptosis. Cell.

[23] Probst BL, Liu L, Ramesh V, Li L, Sun H, Minna JD, Wang L (2010). Smac mimetics increase cancer cell response to chemotherapeutics in a TNF-alpha-dependent manner. Cell Death Differ.

[24] Lecis D, Drago C, Manzoni L, Seneci P, Scolastico C, Mastrangelo E, Bolognesi M, Anichini A, Kashkar H, Walczak H, Delia D (2010). Novel SMAC-mimetics synergistically stimulate melanoma cell death in combination with TRAIL and Bortezomib. Br J Cancer.

[25] Lecis D, Mastrangelo E, Belvisi L, Bolognesi M, Civera M, Cossu F, De Cesare M, Delia D, Drago C, Manenti G, Manzoni L, Milani M, Moroni E (2012). Dimeric Smac mimetics/IAP inhibitors as in vivo-active pro-apoptotic agents. Part II: Structural and biological characterization. Bioorg Med Chem.

[26] Lecis D, De Cesare M, Perego P, Conti A, Corna E, Drago C, Seneci P, Walczak H, Colombo MP, Delia D, Sangaletti S (2013). Smac mimetics induce inflammation and necrotic tumour cell death by modulating macrophage activity. Cell Death Dis.

[27] Di Nicolantonio F, Arena S, Gallicchio M, Zecchin D, Martini M, Flonta SE, Stella GM, Lamba S, Cancelliere C, Russo M, Geuna M, Appendino G, Fantozzi R (2008). Replacement of normal with mutant alleles in the genome of normal human cells unveils mutation-specific drug responses. Proc Natl Acad Sci U S A.

[28] Manzoni L, Belvisi L, Bianchi A, Conti A, Drago C, de Matteo M, Ferrante L, Mastrangelo E, Perego P, Potenza D, Scolastico C, Servida F, Timpano G (2012). Homo- and heterodimeric Smac mimetics/IAP inhibitors as in vivo-active pro-apoptotic agents. Part I: Synthesis. Bioorg Med Chem.

[29] Ganten TM, Koschny R, Sykora J, Schulze-Bergkamen H, Buchler P, Haas TL, Schader MB, Untergasser A, Stremmel W, Walczak H (2006). Preclinical Differentiation between Apparently Safe and Potentially Hepatotoxic Applications of TRAIL Either Alone or in Combination with Chemotherapeutic Drugs. Clin Cancer Res.

[30] Meerbrey KL, Hu G, Kessler JD, Roarty K, Li MZ, Fang JE, Herschkowitz JI, Burrows AE, Ciccia A, Sun T, Schmitt EM, Bernardi RJ, Fu X (2011). The pINDUCER lentiviral toolkit for inducible RNA interference in vitro and in vivo. Proc Natl Acad Sci U S A.

[31] Arena S, Isella C, Martini M, de Marco A, Medico E, Bardelli A (2007). Knock-in of oncogenic Kras does not transform mouse somatic cells but triggers a transcriptional response that classifies human cancers. Cancer Res.

[32] Roulston A, Muller WJ, Shore GC (2013). BIM, PUMA, and the achilles' heel of oncogene addiction. Sci Signal.

[33] Farmer H, McCabe N, Lord CJ, Tutt AN, Johnson DA, Richardson TB, Santarosa M, Dillon KJ, Hickson I, Knights C, Martin NM, Jackson SP, Smith GC (2005). Targeting the DNA repair defect in BRCA mutant cells as a therapeutic strategy. Nature.

[34] Elgendy M, Sheridan C, Brumatti G, Martin SJ (2011). Oncogenic Ras-induced expression of Noxa and Beclin-1 promotes autophagic cell death and limits clonogenic survival. Mol Cell.

[35] Guegan JP, Ezan F, Theret N, Langouet S, Baffet G (2013). MAPK signaling in cisplatin-induced death: predominant role of ERK1 over ERK2 in human hepatocellular carcinoma cells. Carcinogenesis.

[36] Vantaggiato C, Formentini I, Bondanza A, Bonini C, Naldini L, Brambilla R (2006). ERK1 and ERK2 mitogen-activated protein kinases affect Ras-dependent cell signaling differentially. J Biol.

[37] Janku F, Wheler JJ, Naing A, Stepanek VM, Falchook GS, Fu S, Garrido-Laguna I, Tsimberidou AM, Piha-Paul SA, Moulder SL, Lee JJ, Luthra R, Hong DS (2012). PIK3CA mutations in advanced cancers: characteristics and outcomes. Oncotarget.

[38] McCubrey JA, Steelman LS, Chappell WH, Abrams SL, Franklin RA, Montalto G, Cervello M, Libra M, Candido S, Malaponte G, Mazzarino MC, Fagone P, Nicoletti F (2012). Ras/Raf/MEK/ERK and PI3K/PTEN/Akt/mTOR cascade inhibitors: how mutations can result in therapy resistance and how to overcome resistance. Oncotarget.

[39] Belmont PJ, Jiang P, McKee TD, Xie T, Isaacson J, Baryla NE, Roper J, Sinnamon MJ, Lee NV, Kan JL, Guicherit O, Wouters BG, O'Brien CA (2014). Resistance to dual blockade of the kinases PI3K and mTOR in KRAS-mutant colorectal cancer models results in combined sensitivity to inhibition of the receptor tyrosine kinase EGFR. Sci Signal.

[40] Misale S, Arena S, Lamba S, Siravegna G, Lallo A, Hobor S, Russo M, Buscarino M, Lazzari L, Sartore-Bianchi A, Bencardino K, Amatu A, Lauricella C (2014). Blockade of EGFR and MEK intercepts heterogeneous mechanisms of acquired resistance to anti-EGFR therapies in colorectal cancer. Sci Transl Med.

[41] Prahallad A, Sun C, Huang S, Di Nicolantonio F, Salazar R, Zecchin D, Beijersbergen RL, Bardelli A, Bernards R (2012). Unresponsiveness of colon cancer to BRAF(V600E) inhibition through feedback activation of EGFR. Nature.

