# Supplementary material for: Oncogenic KRAS sensitizes premalignant, but not malignant cells, to Noxa-dependent apoptosis through the activation of the MEK/ERK pathway
Source: Oncotarget. 2015 Mar 12;6(13):10994–1008. doi: 10.18632/oncotarget.3552 (PMC4484434; doi:10.18632/oncotarget.3552)
Supplement: Supplementary file 1 [file oncotarget-06-10994-s001.pdf]

Oncogenic KRAS sensitizes premalignant, but not malignant cells, to Noxa-dependent apoptosis through the activation of the MEK/ERK pathway

Supplementary Material

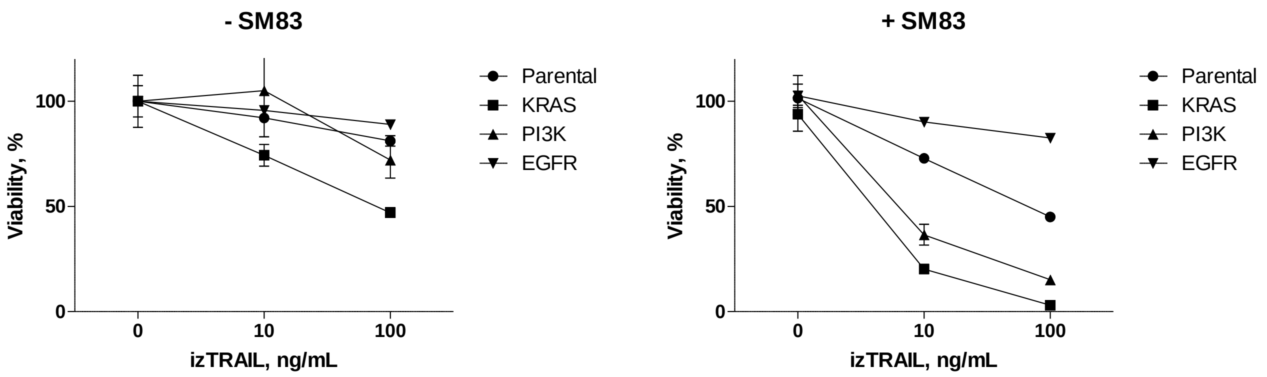

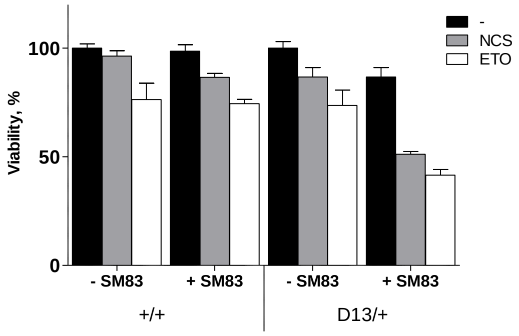

S1: ***Oncogenic KRAS sensitizes HME cells to TRAIL, neocarzinostatin and etoposide.*** The parental human mammary epithelial (HME) cell line and the isogenic cell lines with KI mutations in KRAS (G13D), PI3K (H1047R) and EGFR (delE746A750) were treated with varying doses of izTRAIL alone (**upper left panel**) or in combination with 100 nM SM83 (**upper right panel**) and with 8.8 nM neocarzinostatin (NCS) and 1 µM etoposide (ETO), with or without 100 nM SM83 (**lower panel**). Viability was tested after 24 h. One representative of two independent experiments is shown.

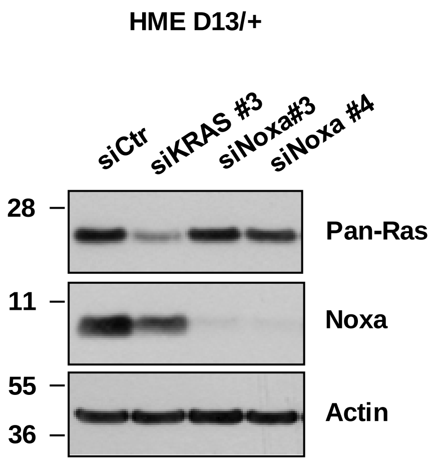

S2: ***Silencing of KRAS down-regulated Noxa levels.*** HME cells bearing the G13D KI mutation were transfected with siRNAs targeting KRAS and Noxa for 72 h. Western blots were performed to evaluate Noxa and pan-RAS levels. Actin is shown as loading control.

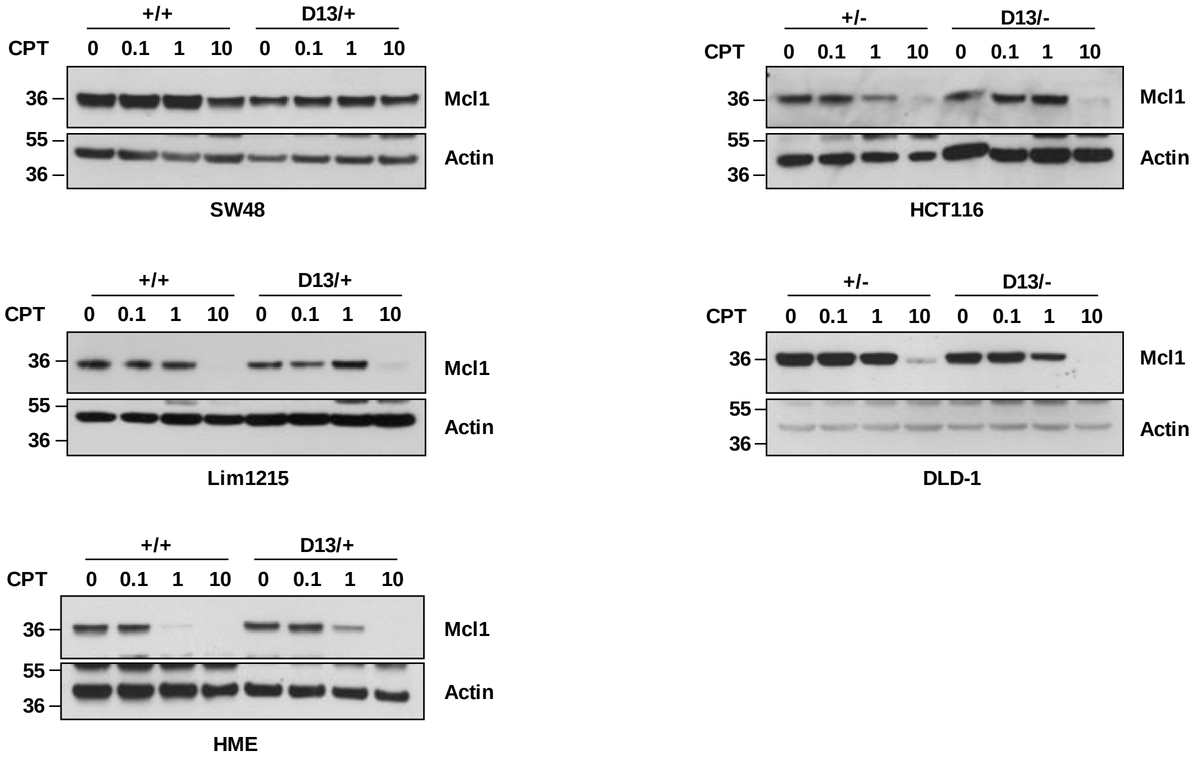

S3: ***Noxa antagonist Mcl1 is more stable in colorectal treated with CPT, compared to HME cells.*** SW48, HCT116, Lim1215, DLD1 colorectal and mammary epithelial HME cell lines were treated with increasing levels of CPT for 6 h. Western blots were performed to detect Mcl1 levels. Actin is shown as loading control.

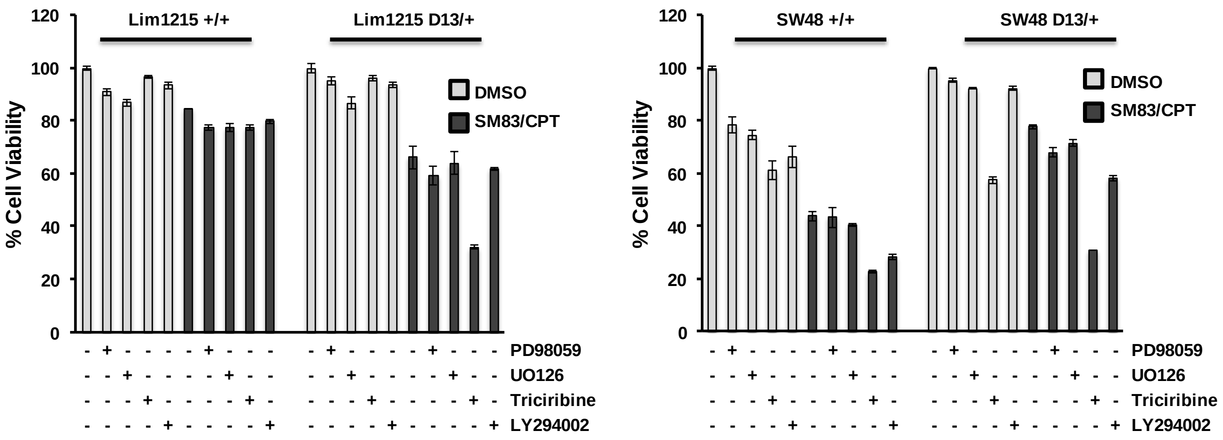

S4: ***AKT activation protects cancer cells from the pro-death effect of oncogenic KRAS even in absence of mutated PI3K.*** Lim1215 (**Left**) and SW48 (**Right**) cells were pre-incubated with 50 µM PD98059, 25 µM UO126, 20 µM Triciribine and 20 µM LY294002 for 2 h, and then mock-treated or treated with 100 nM SM83 and 1 µM CPT. Cell viability was quantified after 24 h. One representative of three independent experiments is shown.
